# High AST/ALT Ratio Is Associated with Cardiac Involvement in Acute COVID-19 Patients

**DOI:** 10.3390/medicina59061163

**Published:** 2023-06-16

**Authors:** Mesut Karatas, Nursen Keles, Kemal Emrecan Parsova, Hatice Ozge Ciftci, Sercin Ozkok, Erkan Kahraman, Furkan Durak, Cevdet Ugur Kocogullari, Nurettin Yiyit

**Affiliations:** 1 Department of Cardiology, Kartal Kosuyolu Yuksek Ihtisas Training and Research Hospital, University of Health Sciences, Istanbul 34865, Turkey; 2 Department of Cardiology, Dr. Siyami Ersek Thoracic and Cardiovascular Surgery Training and Research Hospital, University of Health Sciences, Istanbul 34668, Turkey; 3 Department of Cardiology, Medicana Atasehir Hospital, İstanbul 34750, Turkey; 4 Department of Radiology, Sancaktepe Şehit Prof. Dr. İlhan Varank Training and Research Hospital, University of Health Sciences, Istanbul 34785, Turkey; ozgekass@hotmail.com; 5 Department of Radiology, Acıbadem International Hospital, Istanbul 34149, Turkey; sercinbas2005@gmail.com; 6 Biomedical Science and Engineering, Koc University, Istanbul 34450, Turkey; 7 Department of Cardiology, University of Health Sciences Sancaktepe Şehit Prof. Dr. İlhan Varank Training and Research Hospital, Istanbul 34785, Turkey; 8 Department of Cardiovascular Surgery, Dr. Siyami Ersek Thoracic and Cardiovascular Surgery Training and Research Hospital, University of Health Sciences, Istanbul 34668, Turkey; 9 Department of Thoracic Surgery, Basaksehir Cam & Sakura City Hospital, Istanbul 34480, Turkey

**Keywords:** COVID 19, AST/ALT ratio, De Ritis ratio, cardiac magnetic resonance imaging, cardiac imaging, speckle tracking echocardiography

## Abstract

*Background and Objectives:* We aimed to assess the effect of AST/ALT ratio on echocardiographic and cardiac magnetic resonance imaging (CMRI) parameters after COVID-19 patients recover. *Materials and Methods:* 87 patients with COVID-19 were included in the study. The patients were hospitalized with COVID-19 pneumonia, but the patients did not need intensive care unit follow-up or non-invasive mechanical ventilation support. After a discharge and two weeks following the positive swab test result, patients were considered eligible if they had any symptoms. Transthoracic echocardiography (TTE) was performed within 24 h prior to CMRI. The median value of AST/ALT ratio was found, and the study population was divided into two subgroups based on the median AST/ALT ratio value. The clinical features, blood test, TTE and CMRI results were compared between subgroups. *Results:* C-reactive protein, D-dimer and fibrinogen were found to be significantly higher in patients with high AST/ALT ratio. LVEF, TAPSE, S’, and FAC were significantly lower in patients with high AST/ALT ratio. LV-GLS were significantly lower in patients with high AST/ALT ratio. In CMRI, native T1 mapping signal, native T2 mapping signal and extracellular volume raised significantly in patients with high AST/ALT ratio. Right ventricle stroke volume and right ventricle ejection fraction were significantly lower in patients with high AST/ALT ratio, but right ventricle end systolic volume was significantly higher in patients with high AST/ALT ratio. *Conclusion:* High AST/ALT ratio is related to impaired right ventricular function parameters with CMRI and echocardiography after recovery from acute COVID-19. Assessment of AST/ALT ratio at hospital admission may be used to assess the risk of cardiac involvement in COVID-19 disease, and these patients may require closer follow-up during and after the course of COVID-19.

## 1. Introduction

Coronavirus disease 2019 (COVID-19) is a contagious infectious disease caused by the severe acute respiratory syndrome coronavirus 2 (SARS-CoV-2). It first appeared in China in 2019, and then quickly spread all over the world, and reached the level of pandemic. COVID-19 still remains a major cause of morbidity and mortality worldwide. The main clinical manifestation of COVID-19 is respiratory. COVID-19 respiratory system involvement can vary from mild flu-like presentation to severe symptoms such as viral pneumonia and acute respiratory distress syndrome (ARDS) [1,2,3,4]. On the other hand, COVID-19 may exacerbate underlying chronic cardiac pathologies or lead to the development of new cardiac complications. Case studies have demonstrated that myocardial injury, myocarditis, acute coronary syndrome (ACS), cardiac arrhythmia, heart failure, pulmonary venous thromboembolism may develop in COVID-19 patients [1,2,4,5,6,7]. There are many case reports and reviews reporting cases of viral myocarditis related to COVID-19. Cardiac magnetic resonance imaging (CMRI) is the gold standard diagnostic method for the diagnosis of myocarditis. CMRI with T1 and T2 mapping detects signs of myocarditis such as inflammation, edema, and fibrosis. CMRI provides the evaluation of cardiac structures and functions, and fibrosis can be detected with late gadolinium enhancement (LGE) [8,9,10,11].

Severe pneumonia and respiratory failure in patients with COVID-19 can present as an ARDS. ARDS can cause diffuse alveolar damage, edema, and fibrosis [12,13,14]. Right ventricular dysfunction may develop in moderate and severe ARDS due to pulmonary hypertension as a result of pulmonary thrombosis in the pulmonary microvessels, hypoxic pulmonary vasoconstriction, hypercapnia, and invasive ventilation with high driving pressure [15,16,17].

Fernando De Ritis established the importance of the ratio of serum aspartate transaminase (AST) and alanine transaminase (ALT) (De Ritis ratio) fifty-five years ago. AST and ALT are commonly used biomarkers of liver diseases [18]. While ALT is a more liver-specific enzyme, AST is also commonly found in non-hepatic tissues such as heart and skeletal muscle. Based on the fact that hepatic dysfunction may accompany cardiovascular (CV) diseases, studies have shown that AST/ALT ratio can predict morbidity and mortality in acute and chronic CV diseases [19,20,21,22,23]. Studies have shown that AST/ALT ratio may have prognostic significance in COVID-19 patients [24,25,26]. However, COVID-19 is a systemic disease, and it can affect many tissues such as lung, liver, and heart. Comprehensive cardiac imaging methods are needed to show that the change in AST/ALT ratio may be due to cardiac involvement. In previous studies conducted in COVID-19 patients, no evaluation in this direction has been performed.

Changes in AST and ALT values and ratios, which are frequently evaluated in routine clinical practice, with cardiac diseases has been shown in many studies. Evaluation of the change in AST/ALT ratio during hospitalization and its potential ability to predict cardiac morbidity and mortality may be important to affect the follow-up of patients. In this study, we aimed to assess the effect of AST/ALT ratio at hospital admission on echocardiographic and CMRI parameters after patients with COVID-19 recover.

## 2. Materials and Methods

### 2.1. Study Design and Study Population

This is a prospective observational cohort study. The study was carried out between April 2020 and June 2020. Patients who tested positive for SARS-CoV-2 using reverse transcription-polymerase chain reaction on an upper respiratory tract swab tests were included in the study. The patients had no history of chronic illness, coronary artery disease, heart failure, or more than mild valvular heart disease, or congenital heart disease. The patients were hospitalized with COVID-19 pneumonia, but the patients did not need intensive care unit follow-up or did not need non-invasive mechanical ventilation support. After a discharge and two weeks following the positive swab test result, patients were considered eligible if they had resolved respiratory symptoms, did not have any cardiac symptoms, and had negative swab test results at the end of the isolation period. Patients were not involved the study if they had contraindications for a CMRI. CMRI was performed 4 weeks after the end of the isolation period. Transthoracic echocardiography (TTE) was performed within 24 h prior to CMRI. Clinical features and laboratory test results were obtained at the first hospital admission. The median value of AST/ALT ratio was found, and the study population was divided into two subgroups based on the median AST/ALT ratio value. The subgroups were named as high AST/ALT ratio and low AST/ALT ratio. The clinical features, blood test, TTE and CMRI results of these two subgroups were compared. The local ethics committee approved the study. All participants provided informed consent.

### 2.2. Echocardiography

Echocardiographic examinations were performed by two cardiologists with at least 15 years of experience in advanced echocardiographic techniques such as speckle tracking echocardiography. TTE was performed with a GE Vivid E95 echocardiography equipment (GE Healthcare; Vingmed Ultrasound, Horten, Norway) with a M5S probe (frequency range: 1.5–4.6 MHz). Single-lead electrocardiogram was obtained during the echocardiographic examination. On the parasternal long-axis view in M-mode, the left ventricle end-diastolic diameter (LVEDD), left ventricle end-systolic diameter (LVESD), end-diastolic interventricular septal (IVS), and posterior wall (PW) thicknesses were obtained. The modified biplane Simpson technique was used to calculate the left ventricular ejection fraction (LVEF). The early diastolic peak flow velocity (E), late diastolic peak flow velocity (A), and E wave deceleration time were determined using transmitral Doppler imaging. Pulsed-wave (PW) Doppler mode was utilized for tissue Doppler imaging (TDI). The temporal velocity integral of the cardiac systolic (Sm) wave as well as diastolic function parameters including mitral inflow early diastolic tissue velocity (Em) and mitral inflow late diastolic tissue velocity (Am) were obtained. The tricuspid annular plane systolic excursion (TAPSE) was obtained from the apical four-chamber view by M mode. S’ was obtained by placing the tissue Doppler on the lateral corner of the tricuspid annulus. Right ventricular fractional area change (FAC) is the area difference between right ventricle (RV) end-diastolic and end-systolic areas measured through RV focused apical view.

The vendor-independent software system (EchoPAC version 202) was used to quantify systolic strain parameters using 2D speckle tracking analysis. Global longitudinal strain (GLS) is the average maximal strain across all 17 myocardium segments, expressed as absolute values. The greater the tissue deformation during systole, the greater the systolic strain value. GLS was computed from the apical 4-chamber, apical 2-chamber, and apical long axis using the semi-automated auto strain 3P endocardial boundary tracking method. Maximum systolic pressure was determined using the duration of aortic valve closure (end-systole). The time interval between the onset of the R-wave on the electrocardiogram (ECG) and the conclusion of the left ventricle (LV) outflow tract signal was estimated using pulsed wave (PW) Doppler.

### 2.3. Cardiac Magnetic Resonance Imaging

CMRI were performed on a 1.5-T MR scanner (Signa Explorer; GE Medical Systems, Milwaukee, WI, USA) with a 32-channel phased-array abdominal coil and electrocardiographic gating. Intravenous sedation was not administered during the examination. All patients were trained how to take a breath before the examination. All examinations were carried out by two technologists and ten years experienced radiologist and cardiologist. Sagittal, coronal, and axial localizers through thorax revealed by steady-state free precession sequence and axial stack of black blood using fast spin echo for imaging of cardiothoracic anatomy first. Than, short-axis and long-axis cine balanced steady-state free precession cine movie sequence was performed for cardiac function, and volume. Each set of images was acquired with retrospective gating and 20 reconstructed cardiac phases. Tissue characterization of the LV was performed with T1 mapping using long-T1 5(3)3 shortened modified look locker inversion recovery, and T2 mapping using T2-prepared balanced steady-state free precession. Then, LGE imaging (phase-sensitive inversion-recovery sequence) was performed after administration of the gadolinium-based contrast agent (0.2 mmol/kg). Post-contrast T1 mapping sequence was revealed after 5 min of contrast agent injection, and late gadolinium enhancement sequence was revealed for imaging of myocardial scar after 10 and 15 min of contrast agent injection. All sequences were performed on three short-axis slices from the base, middle, and apical LV.

All CMRI were reviewed by a ten-year experienced radiologist and a fifteen-year experienced cardiologist. CMRI analysis for LV volumes and function was performed using Circle cvi42 software (Circle Cardiovascular Imaging, Calgary, Canada). LV and RV volume/function parameters were automatically calculated by contouring endocardial and epicardial contours. LGE images was assessed visually. For native, post-contrast T1 mapping and T2 mapping, the endocardium and epicardial contouring was performed for the basal, midventricular, and apical segment of short-axis slices. Blood pool contamination was avoided during contouring the epicardial and endocardial borders.

### 2.4. Statistical Analysis

IBM SPSS Statistics version 25 was used for statistical analysis (IBM Corp, Armonk, NY, USA). Nominal variables are expressed as numbers and percentages, whereas continuous variables are expressed as mean and standard deviation. The chi-squared test was used to compare groups for nominal variables, the Student’s *t*-test for continuous variables with normal distribution, and the Mann–Whitney U test for continuous variables without normal distribution. Statistical significance was defined as a *p* value less than 0.05.

## 3. Results

Eighty-seven patients (mean age 42.0 ± 9.9 years and 62.2% male) were involved in the study. Patients were divided into two subgroups depending on the median AST/ALT ratio: those with low AST/ALT ratio (*n* = 43 patients) and those with high AST/ALT ratio (*n* = 44 patients). The median AST/ALT ratio was 0.69. Baseline clinical features, and laboratory test results of patients were shown in Table 1. Baseline patients’ characteristics were comparable between the study subgroups. Hemoglobin, total protein, C-reactive protein, D-dimer, and fibrinogen were found to be significantly higher in patients with high AST/ALT ratio (12.6 ± 2.32 vs. 13.7 ± 2.0 *p* = 0.037, 66.9 ± 4.7 vs. 75.2 ± 3.6 *p* < 0.001, 7.5 ± 34.1 vs. 13.9 ± 24.4 *p* < 0.001, 0.28 ± 0.15 vs. 1.05 ± 0.72 *p* < 0.001, 406.9 ± 93.2 vs. 655.5 ± 102.3 *p* < 0.001, respectively). AST was significantly lower in patients with low AST/ALT ratio (22.8 ± 6.5 vs. 64.4 ± 43.1 *p* < 0.001). Other laboratory test results did not differ between study subgroups.

Echocardiography results of the patients were provided in Table 2. Left atrial maximum volume (LAV max) were significantly larger in the patients with high AST/ALT ratio (27.3 ± 12.1 vs. 44.7 ± 8.3 *p* < 0.001). LVEF, TAPSE, S’, and FAC were significantly lower in patients with high AST/ALT ratio (63.9 ± 4.1 vs. 58.9 ± 3.0 *p* < 0.001, 25.3 ± 3.8 vs. 22.4 ± 2.4 *p* < 0.001, 0.18 ± 0.0.5 vs. 0.12 ± 0.03 *p* < 0.001, 41.9 ± 7.6 vs. 34.8 ± 1.5 *p* < 0.001, respectively). LV 4-chamber longitudinal strain (LS), LV 2-chamber LS, LV 3-chamber LS, and LV-GLS were significantly lower in patients with high AST/ALT ratio (−19.2 ± 1.9% vs. −16.8 ± 1.2% *p* < 0.001, −20.7 ± 2.8% vs. −17.9 ± 2.1% *p* < 0.001, −18.9 ± 2.6% vs. −16.9 ± 1.4% *p* < 0.001, −19.4 ± 1.7% vs. −17.5 ± 1.0% *p* < 0.001, respectively). Other echocardiographic parameters did not differ between the study subgroups.

In CMRI, native T1 mapping signal, native T2 mapping signal and extracellular volume (ECV) raised significantly in patients with high AST/ALT ratio (1034.59 ± 48.60 vs. 1086.49 ± 32.38 *p* < 0.001, 81.61 ± 23.11 vs. 99.95 ± 18.09 *p* = 0.001, 17.3 ± 5.0 vs. 36.2 ± 14.0 *p* < 0.001, respectively). Right ventricle stroke volume (RVSV) and right ventricle ejection fraction (RVEF) were significantly lower in patients with high AST/ALT ratio (55.11 ± 8.75 vs. 48.01 ± 4.97 *p* < 0.001, 55.11 ± 8.75 vs. 48.01 ± 4.97 *p* < 0.001, respectively), but right ventricle end systolic volume (RVESV) was significantly higher in patients with high AST/ALT ratio (63.37 ± 15.98 vs. 74.91 ± 18.74 *p* = 0.002) (Table 3).

**Figure 1:**
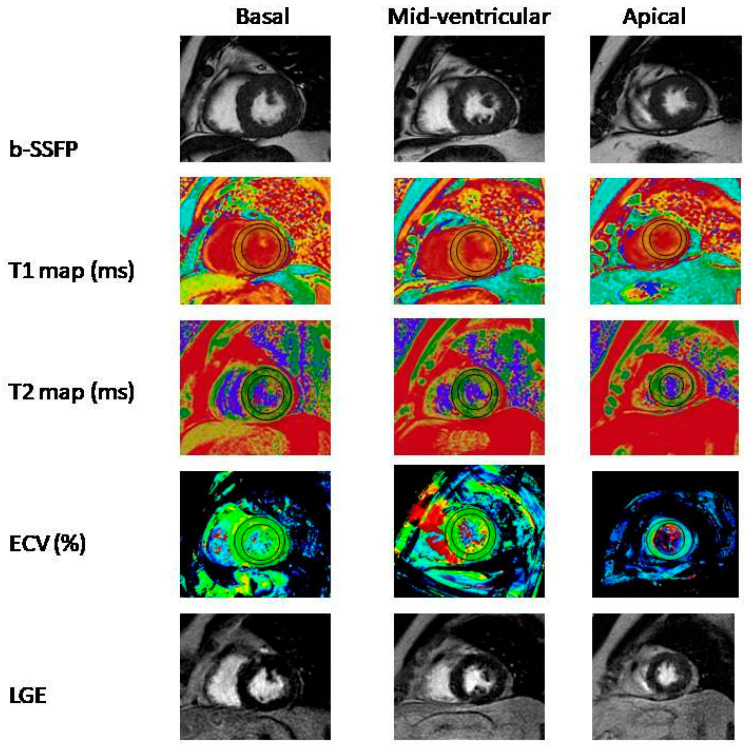
Cardiac MRI examination of a 35-year-old male patient diagnosed with COVID-19. The same slices of short axis view on basal, mid-ventricular, and apical segment (left-to-right) for b-SSFP (b-SSFP, balanced steady-state free precession), T1 mapping, T2 mapping, ECV (extracellular volume) map and LGE (late gadolinium enhancement) images (up-to-down). T1, T2, and ECV mapping values for entire segments were 1129 ± 8 ms, 41 ± 2 ms, and 30 ± 2%, respectively.

LGE was observed in two patients in patients with low AST/ALT ratio group and in four patients in the high AST/ALT ratio group (Figure 1).

## 4. Discussion

The negative effects of the COVID-19 pandemic continue to be seen around the world. COVID-19 can cause complications with direct and indirect effects on the CV system, these adverse effects are especially more pronounced in those with CV disease. Morbidity and mortality are higher in patients with COVID-19 affecting the CV system. Severe cytokine release and cytokine storm caused by COVID-19 cause inflammation at the vascular and myocardial level, and endothelitis, plaque rupture and myocardial infarction, myocardial injury, myocarditis, arrhythmias, venous thromboembolism can develop. Determining the CV involvements caused by COVID-19 during the disease will make a significant contribution to our fight against COVID-19 and our patient follow-ups after recovery [27,28].

The study included 87 patients with COVID-19 pneumonia, but who did not need intensive care unit follow-up or non-invasive mechanical ventilation support. Patients were included to the study at least two weeks after recovery from COVID-19. AST/ALT ratio were calculated through venous blood samples taken during first admission to the hospital. The study group was divided into two subgroups based on the median value of AST/ALT ratio. These two groups were named as high AST/ALT ratio and low AST/ALT ratio. These two groups were compared in terms of acute phase reactants in the course of the disease and CMRI and echocardiography findings. This is the first report to suggest that high AST/ALT ratio may be predict cardiac involvement in patients with COVID-19.

The liver is an important organ that receives approximately one quarter of cardiac output, acts as a reservoir for blood volume, and is extremely sensitive to hemodynamic changes [29,30]. While ALT is mostly found in the liver, AST is found in many tissues such as liver, myocardium, skeletal muscles, erythrocytes, kidney, and brain. Therefore, while ALT mostly reflects liver-specific disorders, AST may also reflect cell death in other tissues. Considering those distribution’s of enzymes, an isolated increase in AST leading to an elevated AST/ALT ratio suggests nonhepatic source. Therefore, the AST/ALT ratio can potentially reflect CV diseases and the associated systemic hypoperfusion [22]. Previous studies have shown that AST/ALT ratio can be used as a predictor in CV diseases. High AST/ALT ratio is associated with long-term mortality after acute myocardial infarction (AMI) [22]. In another study conducted in patients with cardiac arrest, it was shown that high AST/ALT ratio is associated with in-hospital mortality [23]. In studies conducted in patients with heart failure reduced ejection fraction (HFrEF), it was demonstrated that higher AST/ALT ratio was correlated with higher NT-proBNP and could be used as a predictor of functional status in patients with HFrEF [31]. Another study showed that high AST/ALT ratio was associated with poor outcomes within 3 months after acute stroke [20].

Endotheliitis and procoagulant state are common in patients with COVID-19. Interleukin-6 (IL-6) increases with the inflammatory response that develops as a result of COVID-19 pneumonia. The increase in IL-6 leads to endotheliitis and cell damage in liver. In the pathological study, it was shown that COVID-19 increased von Wildebrand factor (vWF) levels, increased platelet accumulation via IL-6-related pathways, and as a result of all, fatty liver cells, increased liver cell damage and increased ALT levels were observed. Inflammatory response also has an important role in the pathophysiology of CV diseases. Systemic inflammation can cause or result from CV events. Elevation of pro-inflammatory cytokines is associated with fatal CV events [32]. On the other hand, SARS-CoV-2 itself may cause liver damage by directly affecting liver cells via angiotensin converting enzyme-2 (ACE-2) receptors. Since ALT is an enzyme that is more sensitive to liver damage and increases more in liver damage, it can be seen that ALT is increased more than AST [32,33,34]. In our study, mild elevation of ALT was found in both patient groups (53.8 ± 19.6 vs. 55.4 ± 26.9, *p* = 0.749), but there was no statistically significant difference between the two study groups. The factor that actually increases the AST/ALT ratio is the elevation of AST. The fact that COVID-19 causes systemic effects and adversely affects many organs may increase the level of AST, which is more common in tissues in the body, than ALT. However, the pathophysiologic mechanisms involved in liver impairment in patients with COVID-19 are still unclear.

It was demonstrated that T1 mapping signals can allow quantitative assessment of myocardial fibrosis, and an increase in native T1 mapping signal indicated myocardial interstitial fibrosis. On the other hand, native T2 mapping signals assessment can accurately detect characterization of myocardial edema, and the early detection of reversible myocardial disease without the use of contrast agents and ionizing radiation, and an increase in native T2 mapping signal indicated myocardial edema [35,36,37]. A previous study included 100 patients with COVID-19 and patients were assessed with CMRI after recovery. CMRI abnormal findings including myocardial native T1 increase, myocardial native T2 increase, myocardial late gadolinium enhancement, or pericardial enhancement were observed in 78% of patients [38]. In our study, native T1 and T2 mapping signal and ECV values were found to be significantly higher in patients with high AST/ALT ratio. Additionally, the acute phase reactants C-reactive protein, D-dimer and fibrinogen were found to be significantly higher in patients with high AST/ALT ratio. Higher acute phase reactants can be a sign of higher inflammatory response. In conclusion, our study demonstrated that diffuse myocardial edema and fibrosis may exist in patients with high AST/ALT ratio.

Recent studies have demonstrated the cardiac effect of COVID-19 after recovery with speckle tracking echocardiography. A recent study included 92 patients who needed hospitalization due to COVID-19. Twenty percent of patients needed intensive care unit follow-up and three patients need mechanical ventilation support. All of the patients had preserved LVEF, but 6.5% of the patients had reduced LV GLS with no alternative etiology [38]. Another recent study with 80 patients with COVID-19 revealed that after three months, all of the patients had preserved LVEF, but 25% of the patients had reduced LV GLS [39]. In our study, it was shown for the first time that LV GLS was reduced in patients with high AST/ALT ratio.

Previous studies have shown that RV impairment can develop in ARDS. SARS-CoV-2’s major target organ is lung, and COVID-19 can induce ARDS, therefore RV may be more susceptible to damage than LV. In ARDS, alveolar injury can develop, on the other hand ARDS can damage the pulmonary circulation via hypoxic pulmonary vasoconstriction, the production of vasoconstrictive mediators, extrinsic vascular compression caused by interstitial edema, and blood vessel remodeling. After that, increased pulmonary vascular resistance, increased right ventricular afterload, and, eventually, RV failure may develop. Myocardial injury and cytokine storm may also impair RV function. Patients with COVID-19 pneumonia may also develop an increase in RV afterload through hypoxia-induced vasoconstriction and inflammatory mediators [11,12,13,14,15,16]. In our study, the patients were hospitalized due to COVID-19 pneumonia but did not develop ARDS and did not need intensive care unit follow-up. In this study, we found that RVESV was higher, while the RVEF and RVSV were significantly lower in the high AST/ALT ratio group in CMRI. In addition, TAPSE, RV S’, and RV FAC, that reflecting echocardiographic RV functions, were also significantly lower in the high AST/ALT ratio group.

### Study Limitations

Our results do not reflect patients with acute COVID-19 infection or those who are entirely asymptomatic when infected with COVID-19. More comprehensive studies are needed to determine a cut-off value for the AST/ALT ratio.

## 5. Conclusions

AST/ALT ratio measurement at the first hospital admission is related to higher acute phase reactants values during the course of COVID-19 infection. High AST/ALT ratio is associated with impaired right ventricular function parameters with T1 and T2 mapping with CMRI and echocardiography after COVID-19 recovered. It also demonstrated for the first time that LV GLS was reduced in patients with high AST/ALT ratio.

It was first reported in this study that high AST/ALT ratio may be predict cardiac involvement in patients with COVID-19. Evaluation of AST ALT ratio at initial presentation may guide the clinician to predict cardiac involvement in patients. Patients with high AST ALT ratios may require closer cardiology follow-up both during and after hospitalization. Studies in which patients will be followed up for a longer period of time may be instructive in terms of the effect of AST ALT ratio in predicting long-term morbidity and mortality.

## Figures and Tables

**Table 1:** Clinical characteristics of the patients, and laboratory test results.

	Low AST/ALT Ratio Group (*n* = 43)	High AST/ALT Ratio Group (*n* = 44)	*p* Value
**Patient characteristics**			
Age (years)	43.7 ± 10.4	40.9 ± 9.3	0.224
Gender			0.599
Male (n, %)	25 (58.1%)	28 (63.6%)	
Female (n, %)	18 (41.9%)	16 (36.4%)	
BMI (kg/m^2^)	26.60 ± 3.73	25.51 ± 4.78	0.304
Systolic arterial pressure (mmHg)	116.5 ± 13.3	119.5 ± 10.6	0.198
Diastolic arterial pressure (mmHg)	75.0 ± 7.2	76.8 ± 7.4	0.340
**Laboratory Data**			
Hemoglobin, g/dL	12.6 ± 2.32	13.7 ± 2.0	**0.037**
Hematocrit (%)	37.3 ± 6.7	39.2 ± 8.1	0.120
White blood cell count (10^3^/μL)	7.7 ± 4.2	7.3 ± 2.5	0.840
Neutrophil (10^3^/μL)	5.4 ± 3.8	5.1 ± 2.5	0.557
Lymphocyte (10^3^/μL)	1.7 ± 0.9	1.7 ± 0.8	0.734
Platelet (10^3^/μL)	310.0 ± 128.2	287.4 ± 94.5	0.376
Serum creatinine (mg/dL)	0.81 ± 0.14	0.87 ± 0.51	0.429
Glucose (mg/dL)	139.8 ± 75.0	151.8 ± 87.5	0.516
Sodium (mEq/L)	134.3 ± 21.7	138.3 ± 2.8	0.260
Potassium (mEq/L)	4.6 ± 0.5	4.5 ± 0.3	0.907
AST (unit/L)	22.8 ± 6.5	64.4 ± 43.1	**<0.001**
ALT (unit/L)	53.8 ± 19.6	55.4 ± 26.9	0.749
AST/ALT	0.46 ± 0.14	1.14 ± 0.41	**<0.001**
Bilirubin (mg/dL)	0.44 ± 0.26	0.40 ± 0.29	0.634
Troponin-T (ng/L)	1.59 ± 4.6	0.71 ± 1.24	0.809
Albumin (g/L)	38.0 ± 7.7	39.3 ± 4.2	0.410
Total Protein (g/L)	66.9 ± 4.7	75.2 ± 3.6	**<0.001**
C-reactive protein (CRP) (mg/dL)	7.5 ± 34.1	13.9 ± 24.4	**<0.001**
D-Dimer (ug/mL)	0.28 ± 0.15	1.05 ± 0.72	**<0.001**
Fibrinogen (mg/dL)	406.9 ± 93.2	655.5 ± 102.3	**<0.001**
Prokalsitonin (ng/mL)	0.04 ± 0.05	0.32 ± 1.34	0.120
Lactate dehydrogenase (LDH) (unit/L)	245.3 ± 60.3	256.5 ± 67.3	0.457
Thyroid stimulating hormone (TSH) (mlU/L)	1.54 ± 0.69	1.67 ± 0.86	0.700

**Table 2:** Echocardiography results of the study group.

	Low AST/ALT Ratio Group (*n* = 43)	High AST/ALT Ratio Group (*n* = 44)	*p* Value
LVEDD (mm)	45.4 ± 2.9	45.4 ± 3.1	0.917
LVESD (mm)	30.3 ± 2.0	30.5 ± 2.7	0.436
LAD-AP (mm)	30.6 ± 4.2	30.9 ± 3.7	0.598
LAV maximum	27.3 ± 12.1	44.7 ± 8.3	**<0.001**
LVEF (%)	63.9 ± 4.1	58.9 ± 3.0	**<0.001**
IVS (mm)	9.5 ± 1.1	9.2 ± 1.1	0.293
PW (mm)	9.4 ± 1.1	9.2 ± 1.1	0.303
E/A ratio	1.17 ± 0.64	1.15 ± 0.55	0.839
Em lateral (cm/s)	0.16 ± 0.05	0.14 ± 0.04	0.097
Am lateral (cm/s)	0.13 ± 0.04	0.12 ± 0.04	0.683
IVRT (ms)	80.5 ± 8.0	83.0 ± 7.3	0.172
IVCT (ms)	75.0 ± 7.3	76.6 ± 9.2	0.683
DT (ms)	180.4 ± 36.8	183.4 ± 38.0	0.466
TAPSE (mm)	25.3 ± 3.8	22.4 ± 2.4	**<0.001**
S’ (cm/s)	0.18 ± 0.05	0.12 ± 0.03	**<0.001**
FAC (%)	41.9 ± 7.6	34.8 ± 1.5	**<0.001**
LV-LS 4 chamber (%)	−19.2 ± 1.9	−16.8 ± 1.2	**<0.001**
LV-LS 2 chamber (%)	−20.7 ± 2.8	−17.9 ± 2.1	**<0.001**
LV-LS 3 chamber (%)	−18.9 ± 2.6	−16.9 ± 1.4	**<0.001**
LV-GLS (%)	−19.4 ± 1.7	−17.5 ± 1.0	**<0.001**

LVEDD, left ventricular end-diastolic diameter; LVESD, left ventricular end-systolic diameter; LAD-AP, left atrium anterior-posterior diameter; LAV; left atrial volume; EF, ejection fraction; IVS, interventricular septum thickness; PW, posterior wall thickness; E, mitral inflow early diastolic velocity; A, mitral inflow late diastolic velocity; Em, mitral inflow early diastolic tissue velocity; Am, mitral inflow late diastolic tissue velocity; IVRT, isovolumic relaxation time; IVCT, the isovolumic contraction time; DT, left ventricular deceleration time; TAPSE, tricuspid annular plane systolic excursion; S’, TDI-derived tricuspid lateral annular systolic velocity wave; FAC, right ventricular fractional area change; LV, left ventricle; LS, longitudinal strain; GLS, global longitudinal strain.

**Table 3:** CMRI results of the study group.

	Low AST/ALT Ratio Group (*n* = 43)	High AST/ALT Ratio Group (*n* = 44)	*p* Value
RVEDV	140.2 ± 25.6	149.6 ± 35.0	0.240
RVESV	63.37 ± 15.98	74.91 ± 18.74	**0.002**
RVSV	55.11 ± 8.75	48.01 ± 4.97	**<0.001**
RVEF	55.11 ± 8.75	48.01 ± 4.97	**<0.001**
T1 MAP Native	1034.59 ± 48.60	1086.49 ± 32.38	**<0.001**
T2 MAP Native	81.61 ± 23.11	99.95 ± 18.09	**0.001**
ECV	17.3 ± 5.0	36.2 ± 14.0	**<0.001**

RVEDV, right ventricle end-diastolic volume; RVESV, right ventricle end systolic volume; RVSV, right ventricle stroke volume; RVEF, right ventricle ejection fraction; ECV, extracellular volume.

## Data Availability

The data that support the findings of this study are available from the corresponding author, (K.E.P.), upon reasonable request.
